# Cytotoxicity by endocrine disruptors through effects on ER Ca^2+^ transporters, aberrations in Ca^2+^ signalling pathways and ER stress

**DOI:** 10.1002/2211-5463.13880

**Published:** 2024-08-13

**Authors:** Francesco Michelangeli, Noor A. Mohammed, Brogan Jones, Monsurat Tairu, Fawaz Al‐Mousa

**Affiliations:** ^1^ Chester Medical School University of Chester UK; ^2^ School of Biosciences University of Birmingham UK; ^3^ Department of Biology University of Duhok Iraq; ^4^ General Directorate of Poison Control Centre Ministry of Health Riyadh Saudi Arabia

**Keywords:** alkylphenols, brominated flame retardants, Ca^2+^ homeostasis, Ca^2+^ transporters, cell death, endocrine disrupting chemicals

## Abstract

Concerns regarding man‐made organic chemicals pervading our ecosystem and having adverse and detrimental effects upon organisms, including man, have now been studied for several decades. Since the 1970s, some environmental pollutants were identified as having endocrine disrupting affects. These endocrine disrupting chemicals (EDC) were initially shown to have estrogenic or anti‐estrogenic properties and some were also shown to bind to a variety of hormone receptors. However, since the 1990s it has also been identified that many of these EDC additionally, have the ability of causing abnormal alterations in Ca^2+^ signalling pathways (also commonly involved in hormone signalling), leading to exaggerated elevations in cytosolic [Ca^2+^] levels, that is known to cause activation of a number of cell death pathways. The major emphasis of this review is to present a personal perspective of the evidence for some types of EDC, specifically alkylphenols and brominated flame retardants (BFRs), causing direct effects on Ca^2+^ transporters (mainly the SERCA Ca^2+^ ATPases), culminating in acute cytotoxicity and cell death. Evidence is also presented to indicate that this Ca^2+^ATPase inhibition, which leads to abnormally elevated cytosolic [Ca^2+^], as well as a decreased luminal ER [Ca^2+^], which triggers the ER stress response, are both involved in acute cytotoxicity.

## Graphical abstract

Acute exposure to a variety of endocrine disrupting chemicals (EDC) such as some alkylphenol and brominated flame retardants (BFRs) can affect ER associated Ca^2+^ Transporters that include ER Ca^2+^ ATPases, to cause exaggerated elevations cytosolic Ca^2+^ levels within cells that leads activation of Ca^2+^‐dependent cell death pathways such as apoptosis and autophagy.

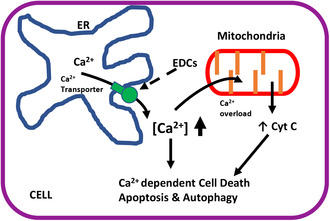

**Abbreviations**
AP: Alkylphenol
BFRs: brominated flame retardants
BHT: butylated hydroxytolulene
BPA: bisphenol‐A
DBBP: dibromobiphenyl
DBPE: decabromodiphenyl ether
EDCs: endocrine disrupting chemicals
HBCD: hexabromocyclododecane
IP_3_
: inositol trisphosphate
LC_50_
: concentration causing 50% cell death
NP: nonylphenol
OBDE: octabromodiphenyl either
PBDE: pentabromodiphenyl ether
POPs: persistent organic pollutants
SERCA: sarcoplasmic/endoplasmic reticulum Ca^2+^ ATPase
TBBPA: tetrabromobisphenol‐A
TBBPA‐DAE: tetrabromobisphenol‐A‐Diallyl ether
tBHQ: tert‐butylhydroxyquinone

Technically, an endocrine disruptor is a chemical that can interact with and change the action of any part of the endocrine system. However, in the early days of their research, it was the term commonly used to describe either man‐made chemicals such as Bisphenol A (BPA) or natural chemicals such as flavonoids, that exhibit estrogenic or anti‐estrogenic activity (which are more correctly called xenoestrogens). In some of the earlier studies investigating man‐made endocrine disruptors the focus was on the potential effects on development and fertility and especially on their effects on sperm quality, which some studies had shown to decrease within the human population over the recent decades [1, 2]. In fact, as early as the 1970s studies were showing that the insecticide such as chlordecone (Kepone), a cyclic aliphatic halogen containing compound, was linked to adverse health effects in humans working in the manufacturing plants making this product. The symptoms exhibited by exposed male workers involved effects on the central nervous system, enlarged liver and both low sperm cell counts and decreased percentages of motile sperm cells [3, 4, 5]. *In vitro* studies additionally confirmed that this insecticide had estrogenic activity, thus indicating a possible mode of action for its toxicity [6]. Further studies investigating a range of other environmental pollutants such as Bisphenol A (BPA) and nonylphenol, used in the plastics industry also confirmed that they could bind directly to oestrogen receptors [7, 8]. Although the initial premise that these man‐made chemicals were acting as estrogenic effectors, other endocrine disrupting functions of these man‐made chemicals have since been identified, such as binding to thyroid hormone receptors [9], as well as alterations in cellular signalling pathways such as Ca^2+^ signalling pathways [10, 11, 12] and MAP kinase pathways [13, 14, 15] which are fundamental to the molecular modes of action that many hormones, cytokines, growth factors and neurotransmitters, use for their physiological effects.

Since these early studies, there have been many other studies to link many man‐made organic chemicals, that persist in the environment with their ability to bioaccumulate within many organisms including man, and potentially cause adverse health effects [16, 17, 18].

This review will give a personal perspective on the substantial body of evidence that has accumulated recently to implicate and explain the endocrine disrupting and cytotoxic effects of some environmental pollutants, via modulation of Ca^2+^ transporters and aberrations in Ca^2+^ signalling pathways, which is fundamental for the action of many hormones and neurotransmitters.

## Endocrine disruptors and their bioaccumulation

To date, a vast number of man‐made substances have been identified as potential endocrine disrupting chemicals. Many of these appear to be widely disbursed within the environment and have been incorporated into food chains [19]. Chemicals that are of some concern regarding human health include; some pesticides; brominated flame retardants (BFRs), such as tetrabromobisphenol‐A (TBBPA) which are ubiquitous in many types of upholstery fabrics and plastics encasing electronic good; phthalates and other plasticizing agents commonly found in plastics, industrial coatings; and detergents, such as Bisphenol A (BPA) and nonylphenol.

**Fig. 1:**
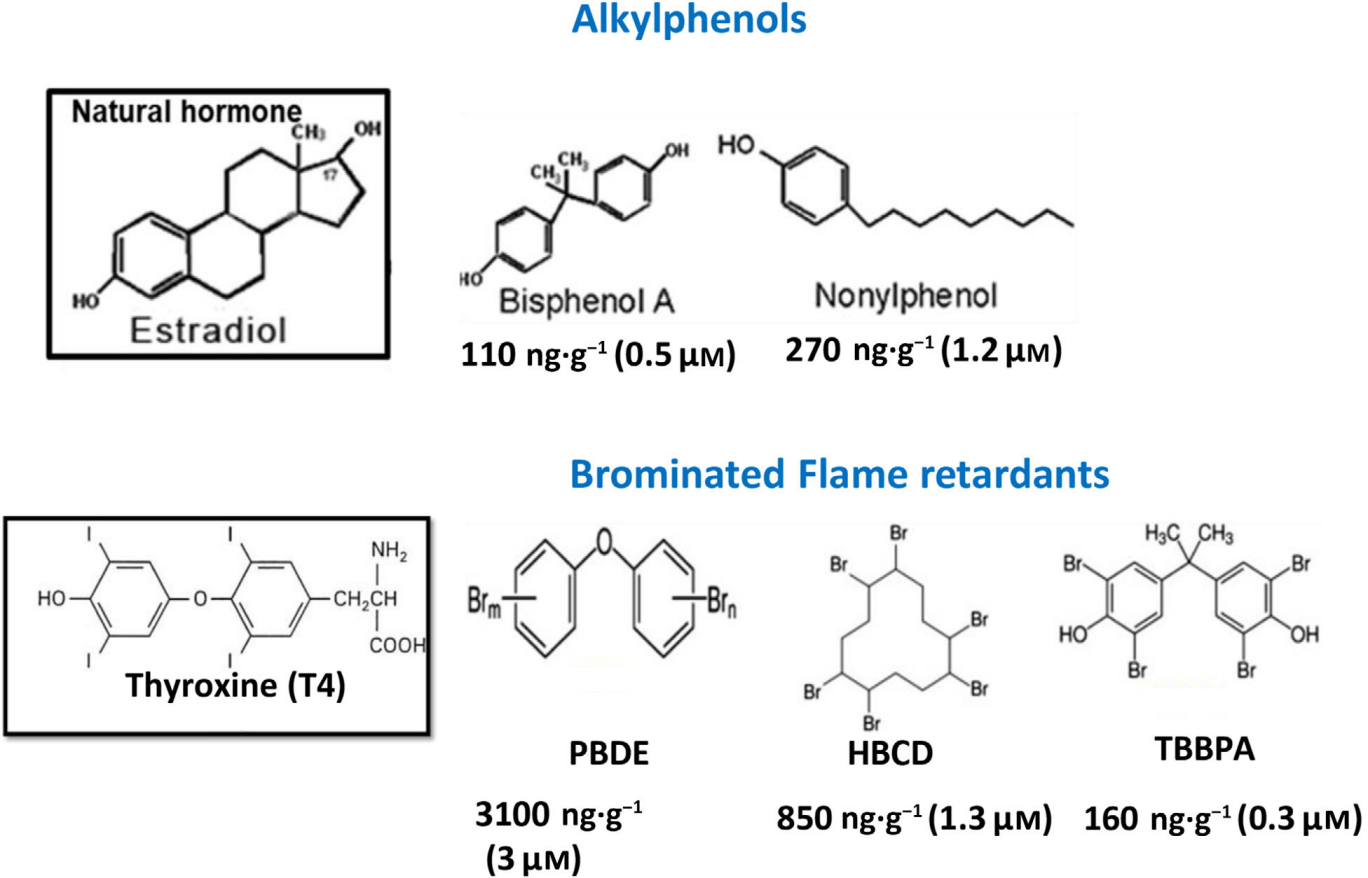
Structures and bioaccumulation levels of some common alkylphenols and BFRs. The figure shows the chemical structures of some common alkylphenols and BFRs focussed on in this review. The structures of estradiol and thyroxine hormones are also included as a comparison. The figures given for each chemical comes from measurements made from human blood samples (values given in ng·g^−1^ lipid weight and equivalent μm concentrations).

Figure 1 shows some examples of EDC that belong to the family of plasticizing agents/coatings as well as some brominated flame retardants, that were widely used until their recent phasing out. As can be seen in Fig. 1, a number of plasticizing agents have several physico‐chemical properties and size similar to oestrogen, while some of the brominated flame retardants (BFRs) appear to also have some chemical characteristics in common with the thyroid hormone, thyroxine, which again strengthens the premise that these chemicals could be acting as hormone agonists or antagonists.

As some of these chemicals do not bio‐degrade readily within the environment they are also termed as persistent organic pollutants (POPs), which have been shown to bioaccumulate into a variety of organisms, which can therefore prolong their ability to affect the ecosystem. In humans, the reported concentrations of some of these chemicals are at levels that could cause adverse health effects (Fig. 1, also indicates the concentration that some of these chemicals have been found in human blood samples). Toxicokinetic studies with BPA and TBBPA have shown varied half‐lives in the body ranging from a few hours (for BPA) to 1–2 days (TBBPA), with the major route of elimination being through glucuronidation and urine excretion. However, a portion of these chemicals were also found to bioaccumulate in adipose tissues and other high fat containing organs and therefore some of these chemicals remained in the body for a much longer time period [18, 19]. The concentration of some of these EDCs has been reported to reach μm levels in human tissues and 10s of μm in fish living in polluted waters [18, 19].

A number of studies have implicated or proposed that some alkylphenols and BFRs may be causing a variety of diseases in man, these have included metabolic disorders such as Obesity, Diabetes Mellitus [20] and Cardiovascular Diseases; Female Reproduction disfunction; Male Reproduction disfunction; Thyroid Disruption; Neurodevelopmental and Neuroendocrine Effects, to list a few [18, 19, 21, 22, 23]. It is clear, therefore, that a detailed understanding into how these chemicals interact with living systems to cause these adverse effects is required. It has also been noted, in *in vitro* and animal studies, that some of these chemicals have the ability to be cytotoxic in nature and therefore a detailed study into how they cause cell disfunction and death is also required.

## Cell death pathways activated by EDC

The molecular mechanisms by which EDC such as alkylphenols (i.e., BPA, bisphenol, nonylphenol) and BFRs such as hexabromocyclododecane (HBCD), tetrabromobisphenol‐A (TBBPA) and polybrominated diphenyl ethers (PBDE) cause cell death through targeting the Ca^2+^ signalling pathway has been extensively studied by our group and others. A large body of evidence has now been gained to show that acute exposure to biologically relevant concentrations (Fig. 1), causes cell death in a variety of cell types (Table 1).

**Table 1:** Effects of EDC on cell viability for a range of cell types and their potency at inducing cell death.

Endocrine disrupting chemical	Cell line and origin	LC_50_ ^a^	References
BFRs			
HBCD	SH‐SY5Y (neuronal)	3 μm	[24]
	RBL2H3 (leukocyte)	1.5 μm	[25]
	HepG2 (hepatocyte)	28 μm	[25]
	Cos‐7 (renal)	17 μm	[25]
TBBPA	SH‐SY5Y (neuronal)	15 μM	[26]
	TM4 (Sertoli‐Testis)	18 μm	[12]
	Cal‐62 (thyroid)	200 μm	[13]
	NRK (kidney)	25 μm	[13]
	A549 (lung)	100 μm	[13]
DBPE (Deca‐Br)	SH‐SY5Y (neuronal)	28 μm	[26]
PBDE(Tetra‐Br)	SH‐SY5Y (neuronal)	16 μm	[24]
AlkylPhenols			
Nonylphenol	SH‐SY5Y (neuronal)	6 μm	[27]
	TM4 (Sertoli‐Testis)	10 μm	[28]
	MC3T3‐E1 (Osteoblast)	20 μm	[29]
BPA	TM4 (Sertoli‐Testis)	300 μm	[10]
	MC3T3‐E1 (osteoblast)	30 μm	[29]
	SH‐SY5Y (neuronal)	183 μm	[30]

^a^ LC_50_, the concentration of the chemical that causes a reduction of 50% cell viability.

Early studies with alkylphenols such as nonylphenol and BPA where focussed on investigating their effects on testis‐derived cells due to their implication with low and/or poor quality, sperm production through Sertoli cell dysfunction and death [1, 2, 10, 31]. With respect to BFRs, some of the early studies focused on their toxicity to neuronal cells as they were shown to cross the blood–brain barrier and cause neurological disorders in cognitive development, in animal studies, causing affects on learning, memory and motor functions [32, 33]. However, since these early studies it has been shown that a variety of alkylphenols and BFRs are able to affect a multitude of different cell types derived from a range of different tissues and organs (see Table 1 for examples). In this respect, a recent comparative study investigating the cytotoxicity of the BFR, HBCD, using identical experimental conditions, showed that although it caused acute toxicity and cell death in a range of cell types ie leukocyte‐derived, neuronal‐derived, liver‐ and kidney‐derived. However, the neuronal and leukocyte cells appeared to be far more sensitive [25]. This suggested that some selectivity to toxicity does occur in different cell types.

Due to the acute cytotoxicity of these EDC in a variety of cell types, further studies were undertaken to determine the molecular mechanism(s) of cell death involved. Many studies with BPA and nonylphenol, as well as BFRs, have indicated that the major mode of cell death is through apoptosis [10, 25, 28]. This was determined through observation of nuclear morphological changes, DNA fragmentation (DNA laddering) and the use of specific caspase inhibitors and fluorogenic caspase substrates [12, 25, 26], as well as observing cytochrome C release from the mitochondria and mitochondrial depolarisation within cells [12, 26]. Apoptosis was also shown to occur during BFRs exposure (such as HBCD, TBBPA and PBDE) in a variety of cell types including in Sertoli cells, macrophage‐like cells and neuronal cells [12, 26].

Furthermore, as both alkylphenols and BFRs were shown to involve caspase‐8 and caspase‐9 [27], it is likely that both intrinsic and extrinsic apoptotic pathways are involved. Although several studies identified that the activation of caspases were involved [12, 26, 27], one study indicated that cell death was caused by atypical apoptosis (independent of caspases) [34].

A recent more extensive study into the mode of cell death by HBCD has identified that other cell death pathways are also activated, in addition to apoptosis. This study identified a possible role for autophagy as shown by the formation of GFP‐tagged LC3 binding to autophagosomes [25] as well a gel mobility shift of LC3 I to LC3II due to lipidation [35]. Similar experiments have also implied that autophagy is involved in cytotoxicity by nonylphenol and BPA [36, 37]. No early onset necrosis was determined by assessing cell membrane permeability, a hallmark for necrosis (via Propidium iodide (PI) staining or Lactate dehydrogenase (LDH) cell leakage measurements) with either HBCD [25] or BPA [36, 38] suggesting that necrosis was unlikely to be involved. An investigation for a role by necroptosis in causing cell death was also investigated via the use of necrostatin‐1 (RIP‐1 kinase inhibitor involved in necroptosis). The experiments in HepG2 cells and SHSY‐5Y cells showed that HBCD‐induced cell death was unaffected by pre‐treatment with necrostatin 1 [25] and therefore necroptosis was also an unlikely mechanism in these cells. Necroptosis was, however, shown to be implicated in BPA toxicity in cardiac‐derived endothelial cells as RIPK‐3, another mediator of necroptosis was upregulated in these cells [39], potentially suggesting that different mechanisms of cell death may be involved in different cell types.

It is known that exaggerated elevation in cytosolic [Ca^2+^] levels can initiate cell death and that buffering this [Ca^2+^] rise can reduce this. Studies with both alkylphenols [10] and HBCD [25] have shown that pre‐treating cells with the membrane‐permeant Ca^2+^ chelating agent (BAPTA‐am) followed by exposure to these chemicals significantly protected the cells from cell death, indicating that, at least in part, cell death was via Ca^2+^‐dependent mechanism(s), activated through the exaggerated elevation in cytosolic [Ca^2+^] levels.

## Molecular basis of abnormal Ca^2^, homeostasis affecting cell viability and cytotoxicity

**Fig. 2:**
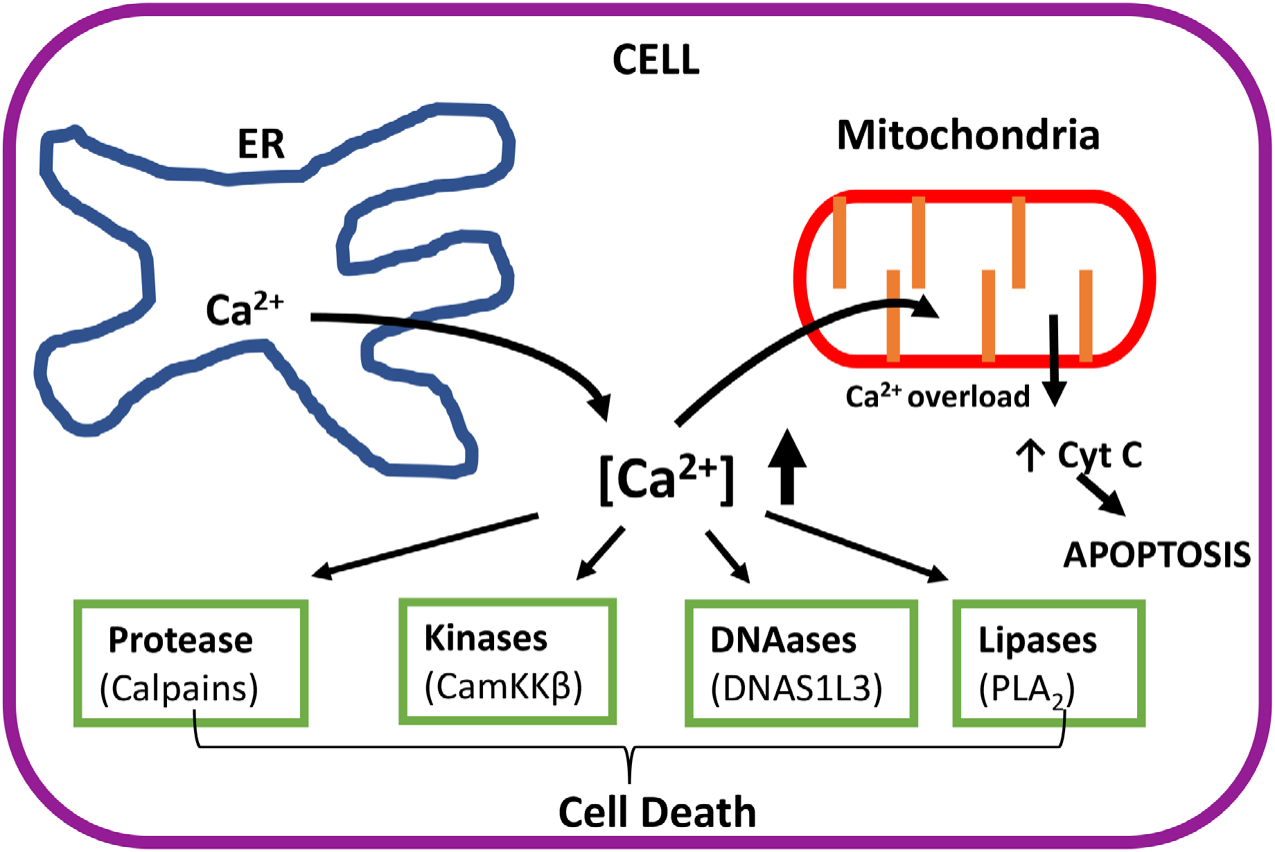
The hyperactivation of key Ca^2+^‐dependent enzymes leading to cell death. The figure illustrates the role of Ca^2+^ release from the ER leading to the exaggerated increase in cytosolic [Ca^2+^], which in turn both hyperactivates key Ca^2+^‐dependent enzymes as well as causing mitochondrial ‘Ca^2+^ overload’ causing activation of apoptosis through the release of cytochrome C. Specific examples of Ca^2+^‐dependent enzymes that are known to contribute to cell death are identified. Calpains, are involved in cytoskeletal protein breakdown and proteolytic modulation of death inducing enzymes [44, 45]; CamKKβ is known to affect AMP‐kinase which leads to mTOR inhibition and autophagic cell death [46]; DNAS1L3 is a Ca^2+^‐dependent DNAase which is involved in DNA fragmentation during apoptosis [47] and PLA_2_ is a lipase involved in arachidonic acid production leading to inflammation and cell death [48].

Moderate elevations in cytosolic [Ca^2+^] levels have been known to be part of normal physiological functions for many years [40]. The requirement for Ca^2+^ and changes of Ca^2+^ concentration during muscle contraction has long been known for more than 140 years. With the advent of cell‐permeant Ca^2+^ sensing dyes in the 1980s, developed by Roger Tsein and co‐workers, it was clearly demonstrated that many physiological processes induced by specific bioactive chemical stimuli which include some hormones, neurotransmitters, pheromones, drugs and even physical and mechanical stimuli, results in short term and modest the transient elevation in intracellular or cytosolic [Ca^2+^] changes [40]. In some cases, these modest changes may occur over longer periods in a controlled oscillatory manner, or be limited to defined areas inside cells [41]. However, it has also been noted for many years, that over stimulation of this Ca^2+^ signalling pathway, for example abnormally raised levels of cytosolic [Ca^2+^] which tends to be temporally more prolonged, has the ability to cause cellular dysfunction and death [42]. The reason for this is multifaceted, but includes excessive Ca^2+^ uptake into the mitochondria from the cytosol causing depolarisation and ‘mitochondrial Ca^2+^ overload’ and subsequent uncontrolled release of Ca^2+^ and mitochondrial proteins such as cytochrome C [26]. This then leads to apoptosis via the intrinsic pathway as well as hyperactivation of numerous Ca^2+^‐dependent enzymes that include proteases, DNAases, lipases, kinases, phosphatases, etc, some of which are known to be involved in several death pathways (see Fig. 2).

In order to maintain Ca^2+^ concentration levels within defined limits, and only allowing appropriate levels of transient [Ca^2+^] elevations that are highly controlled, a host of intracellular Ca^2+^ pumps (Ca^2+^ ATPases), Ca^2+^ channels, Ca^2+^ exchangers and Ca^2+^ buffering proteins are involved [43]. Many of these reside in specific locations and on organelles, to finely tune the propagating Ca^2+^ signal, as well as coordinating interactions between different organellar Ca^2+^ stores [43].

If this tightly orchestrated control of intracellular [Ca^2+^] levels becomes imbalanced this could lead to potentially drastic consequences and therefore chemicals such as some EDCs that can affect Ca^2+^ transporters may lead to such issues (see below). As a point of interest, some natural chemicals such as flavonoids that are also known to have xenoestrogenic properties found in fruit and vegetables have also been shown to affect Ca^2+^ transporters such as Ca^2+^ pumps [49].

## Effects of EDC on ER Ca^2+^ transporters

### Effects of EDC on SERCA type Ca^2+^ ATPases

Due to the hydrophobic physico‐chemical nature of many EDC they have the ability to efficiently bind to biological membranes, and therefore able to bind and penetrate into cells [28, 50]. In the late 1980s and early 1990s, several studies identified several organic chemicals, some of which were commonly used in plastics manufacture, such Bis(2‐hydroxy‐3‐tert‐butyl‐5‐methylphenyl)‐methane (bisphenol), 2,5‐Di(tert‐butyl)‐1,4‐benzohydroquinone (tBHQ) and nonylphenol, to be potent inhibitors of the skeletal muscle sarcoplasmic reticulum (SR) Ca^2+^ATPase and proved useful in helping to elucidate the molecular mechanism of this Ca^2+^ transporter [51, 52, 53]. The contemporaneous identification that some of these chemicals were also acting as EDCs, led us to investigate whether they could also be affecting normal Ca^2+^ signalling pathways through inhibition of Ca^2+^ ATPases in non‐muscle tissues and cells [10]. In 1994, we demonstrated that addition of micromolar levels of bisphenol to HL‐60 cells induced a prolonged transient rise in cytosolic [Ca^2+^] levels that lasted over 600 s, whereas increases in [Ca^2+^] levels with a physiological agonist (ATP) lasted only 60s [51]. The rise in intracellular [Ca^2+^] was dependent upon the dose of bisphenol added to the cells and was also present in the absence of extracellular [Ca^2+^] in the media, indicating that the cytosolic [Ca^2+^] increase was coming from intracellular Ca^2+^ stores [51]. Furthermore, this study also showed that in addition to the skeletal muscle SR Ca^2+^ATPase (isoform SERCA1a), bisphenol could also inhibit non‐muscle ER Ca^2+^ATPase (isoform SERCA2b) as well as the red blood cell plasma membrane Ca^2+^ ATPase (PMCA1), which are all coded for by different genes. Bisphenol, however, did not significantly affect other ion‐transporting ATPases such as the Na^+^/K^+^‐ATPase [51]. A study using other EDC as inhibitors of the SERCA family of Ca^2+^ATPase showed that the potency of nonylphenol and tBHQ were considerably better inhibitors for the non‐muscle SERCA Ca^2+^ATPase isoforms, SERCA2b and SERCA3a [54].

With regards to bisphenol, it has more recently, been shown that it is a significantly more potent inhibitor to the secretory pathway Ca^2+^ATPase (SPCA1) located in the Golgi body within cells than to the SERCA Ca^2+^ ATPases [55].

**Fig. 3:**
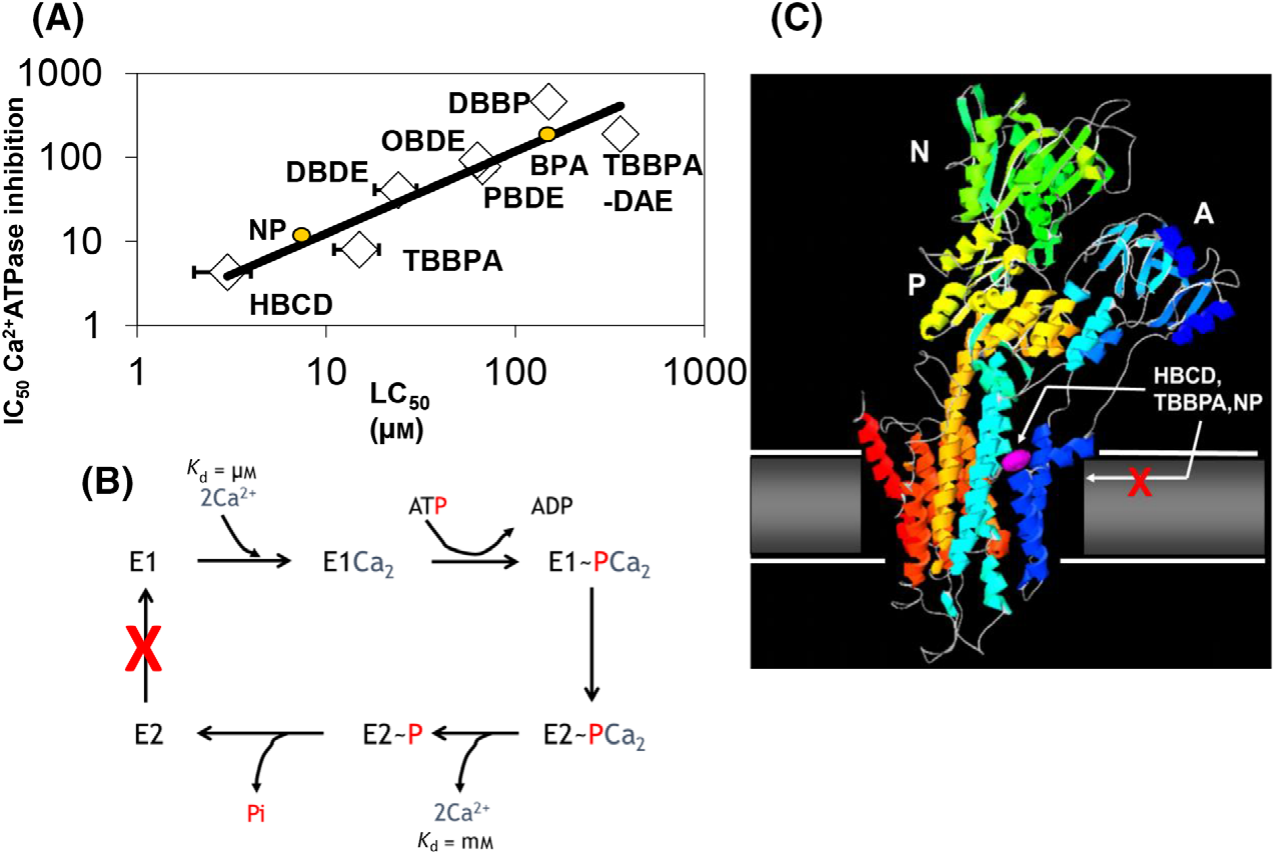
The effects of EDC on the SERCA Ca^2+^‐ATPase and their correlation with cell death. (A) Shows the correlation between the potency of inducing cell death (as studied in the SH‐SY5Y neuronal cells), presented as the LC_50_ value (ie concentration which reduces cell viability by 50%, measured by MTT assay) compared with potency of inhibiting the microsomal Ca^2+^ATPase activity from these cells, for a range of BFRs and alkylphenols. Hexabromocyclododecane (HBCD), Tetrabromobisphenol‐A (TBBPA), Decabromodiphenyl ether (DBPE), Dibromobiphenyl (DBBP), Pentabromodiphenyl ether (PBDE), Octabromodiphenyl either (OBDE), Tetrabromobisphenol‐A‐Diallyl ether (TBBPA‐DAE), Nonylphenol (NP) and Bisphenol‐A (BPA). The correlation coefficient between the 2 variables is 0.94. Data taken from [24]. (B) Show the mechanism of the SERCA Ca^2+^ATPase, highlighting the high (E1) and low (E2) Ca^2+^ affinity binding forms. Also highlighted (by the X) is the E2 going to E1 transition step which is postulated to be the major step affected by alkylphenols and BFRs leading to Ca^2+^ATPase inhibition. (C) Shows the elucidated structure of SERCA1 Ca^2+^ATPase in the E2 conformation and from experimental and modelling studies, it highlights the postulated site at which HBCD, TBBPA and nonylphenol binds.

Over the intervening years our group and others have investigated the effects of a range of alkylphenols and BFRs on; intracellular [Ca^2+^] levels, ER Ca^2+^ATPase activity and their effects on cell viability. Figure 3A, shows a direct correlation between their potency at inhibiting the ER Ca^2+^ATPase (IC_50_ values) and their ability to induce cell death (LC_50_ values), which provides strong evidence for SERCA Ca^2+^ATPase being the molecular target for acute toxicity by some of these EDC [24].

To further investigate the molecular mode of inhibition on the Ca^2+^ATPase by a range of alkylphenols and BFRs, several enzyme kinetic studies were undertaken. The SERCA type Ca^2+^ ATPases undergo a multitude of conformational steps in linking the hydrolysis of ATP to the transport of Ca^2+^, which involves its transient phosphorylation by ATP and alteration in conformation, going from having high‐affinity Ca^2+^ binding sites on one side of the ER membrane (the E1 form), to the lower affinity Ca^2+^ binding form (E2) with the sites on the inside of the ER membrane (Fig. 3B).

In a 1990 study, it was shown that nonylphenol decreased the effective equilibrium constant for phosphorylation of the ATPase by Pi through an increase in the effective rate of dephosphorylation of the phosphorylated ATPase. It also increased the effective equilibrium constant E2/E1 for the ATPase with inhibition of ATPase activity following from the slowing of the E2 → E1 transition [52]. Other studies with the potent SERCA inhibitor Thapsigargin, as well as butylated hydroxytoluene (BHT) and tBHQ, also showed that the E2 to E1 transition was greatly affected with these inhibitors stabilising the low affinity Ca^2+^ binding E2 form [53]. Later studies investigating the mechanism of action of BFRs on the Ca^2+^ATPase also identified that the inhibition by TBBPA and HBCD were again largely through the slowing down of the E2 → E1 transition by stabilising the E2 form [24, 56]. As x‐ray crystal structures of the Ca^2+^ATPase (SERCA1a) have been elucidated including several structures in which inhibitors such as Thapsigargin and tBHQ are bound (PBD code 2AGV, [49, 57]), it is clear that these inhibitors stabilise the Ca^2+^ATPase in an E2 conformation, as also deduced from the mechanistic studies. The structures also show that the sites at which these two inhibitors bind are distinct from each other. Both inhibitors bind at or within the transmembrane domain, with Thapsigargin occupying a site formed from TM3, TM5 and TM7 [49, 57], and tBHQ occupying a site on the opposite side of the transmembrane bundle encompassing M1, M2, M3 and M4, with M1 adopting a kinked conformation potentially allowing tBHQ direct access to this site (Fig. 3C) [49, 57].

The fact that both alkylphenols and BFRs are highly hydrophobic [28, 50] with common EDCs (as measured by oil/water partition coefficient) ranging from a Log *K*_ow_ of 3.6 for BPA to 5.5 for HBCD and 6.2 for nonylphenol. There is a clear correlation between hydrophobicity and potency of Ca^2+^ATPase inhibition for the alkylphenols [28], that these might indicate that these EDC need to partition into the membranes before accessing the Ca^2+^ATPase transmembrane domains causing inhibition. However, several studies have utilised the fact that BFRs contain bromine atoms which have the ability to quench the fluorescence of the tryptophans within the protein (mostly located in the TM region of the Ca^2+^ATPase) by contact quenching mechanisms and therefore can be used to monitor BFR to Ca^2+^ATPase binding interactions [56]. Rapid kinetic experiments were undertaken to measure the rate constants for both tryptophan quenching of the Ca^2+^ATPase by TBBPA and quenching of a fluorophore located in the surrounding lipid bilayer. The results showed that the rate constant was much faster for quenching the intrinsic tryptophans within the Ca^2+^ATPase (*k* = 90 s^−1^) than the rate constant for quenching of the membrane bilayer bound fluorophore (*k* = 0.24 s^−1^), indicating that the BFRs must directly bind to the Ca^2+^ATPase without the need to partition into the lipid bilayer first [56]. In addition, this intrinsic tryptophan fluorescence quenching was also employed with nonylphenol added to the Ca^2+^ATPase that had been reconstituted with brominated phospholipids, which quenches the fluorescence of the tryptophans located at the lipid‐protein interface. As nonylphenol did not reverse the quenching by displacing these brominated phospholipids, it was unlikely that nonylphenol binds at the lipid‐protein interface of the Ca^2+^ATPase [52]. The serendipitous fact that brominated compounds like BFRs can quench the intrinsic tryptophan fluorescence of the Ca^2+^ATPase and therefore can be used as a reporter for BFR binding, was further exploited in order to determine whether some BFRs like TBBPA, bound to the same site on the Ca^2+^ATPase as tBHQ, whose site had already been determined from X‐ray crystallographic studies [57]. The data presented in Ogunbayo and Michelangeli, showed that TBBPA was able to quench some of the Ca^2+^ATPase tryptophan fluorescence and this was reversed by both tBHQ and nonylphenol, indicating that all three inhibitors shared the same binding site on the Ca^2+^ATPase [56]. However, the reversal of TBBPA quenching by thapsigargin was not observed indicating that it bound to a separate/distinct site, as identified from the structural studies [56] (Fig. 3C).

**Fig. 4:**
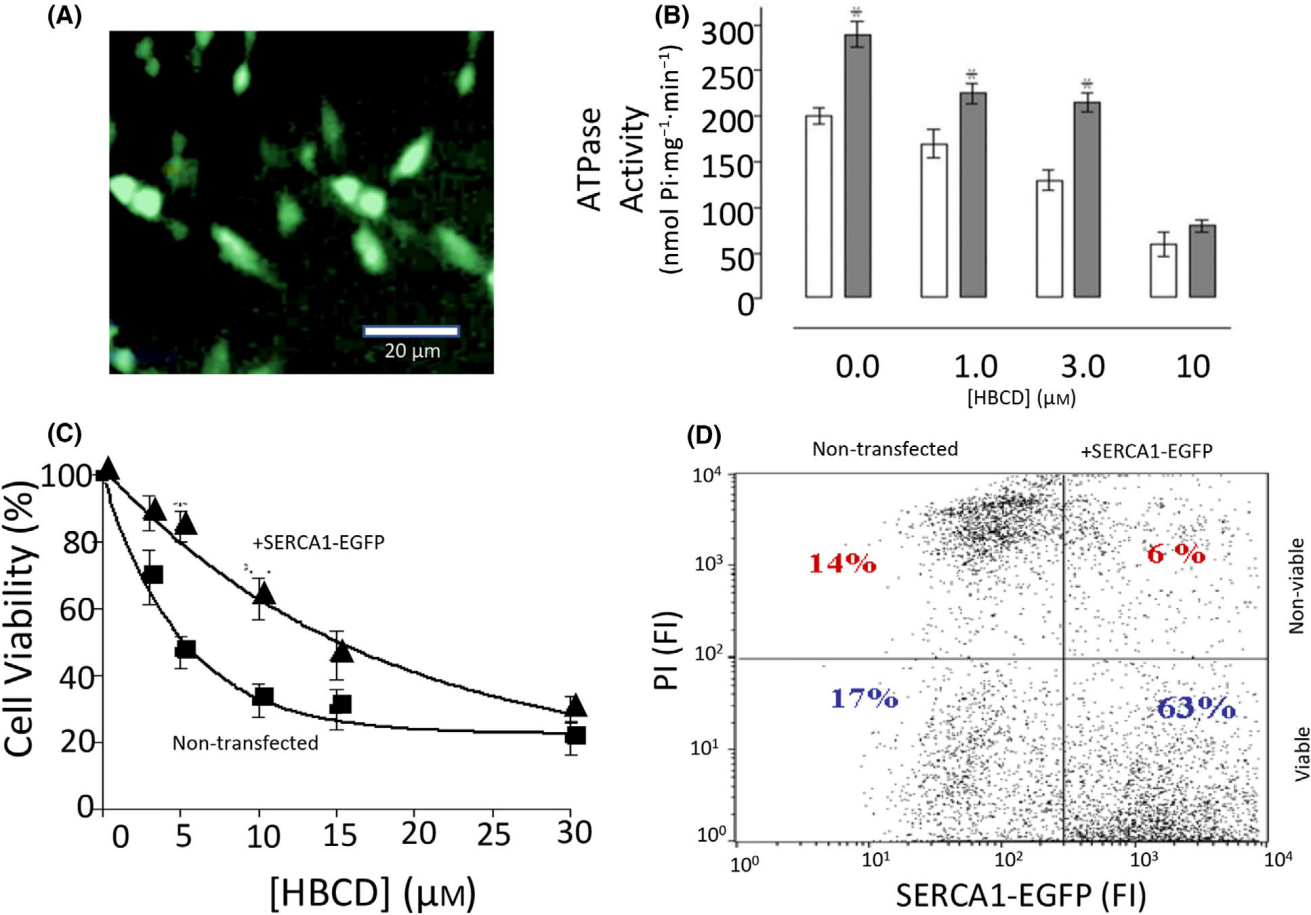
Overexpression of SERCA Ca^2+^ATPase protects against HBCD‐induced cell death. (A) Shows a fluorescence micrograph of SH‐SY5Y cells transfected with SERCA1‐EGFP. The transfection efficiency for these experiments was determined to be between 35% and 70%. (B) Shows the Ca^2+^ATPase activity of microsomes prepared from SH‐SY5Y cells overexpressing SERCA1‐EGFP or non‐transfected. These membranes were exposed to a range of concentrations of HBCD (0–10 μm) and Ca^2+^ATPase activity measured. The data shows that the Ca^2+^ATPase activity is substantially higher for the SERCA1‐EGFP containing microsomal membranes compared to control membranes, over a range of HBCD concentrations. (Data points are the mean of 3 to 4 determinations ± SD, *P < 0.05). (C) Shows the cell viability (measured by MTT assays) of SH‐SY5Y cells, either expressing SERCA1‐EGFP or control cells, when exposed to a range of HBCD concentrations (0–30 μm for 24 h). Data points are the mean ± SD of 4 to 5 determinations. LC_50_ values are 4 ± 1 μm for control cells and 15 ± 2 μm for the SERCA1‐EGFP overexpressing cells. (D) Shows the FACs analysis of SH‐SY5Y cells overexpressing SERCA1‐EGFP and exposed to 3 μm HBCD for 24 h. After incubation with propidium iodide (PI), cells which were non‐viable would have high fluorescence intensities (FI) in the red channel and cells which had overexpression of SERCA1‐EGFP, would have high fluorescence intensities in the green channel. The cells were assigned to four distinct populations. The cells that exhibited high levels of SERCA1‐EGFP fluorescence, showed greater levels of viability when exposed to HBCD compared to the population which did not overexpress SERCA1‐EGFP. In the two populations not overexpressing SERCA1‐EGFP, similar levels of viable to non‐viable cells were measured at 3 μm HBCD. The data presented here were adapted from [27].

In order to more fully explore the premise that the SERCA Ca^2+^ATPase is the major target for acute toxicity by some of these EDC, a further investigation was undertaken to determine whether overexpression of SERCA into cells could protect them from cell death, following exposure to HBCD. In these studies, the neuronal cell line SH‐SY5Y cells were transfected with a green fluorescent protein tagged Ca^2+^ATPase, SERCA1‐EGFP, so that its expression levels in the cells could be monitored and analysed using fluorescent‐activated cell counting (FACs). Studies using the SERCA1‐EGFP pcDNA3.1^+^ plasmid had already shown good levels of expression when transfected into mammalian cells [58]. Figure 4A, shows that once the cells had been efficiently transfected with this plasmid as determined through the presence of green fluorescence, microsomal membranes were prepared from these cells and shown to have increased Ca^2+^‐dependent ATPase activity compared to non‐transfected cell microsomal membranes, thus indicating the SERCA1‐EGFP to be active (Fig. 4B). The membranes from the cells transfected exhibited about 40% higher Ca^2+^ATPase activity than non‐transfected cell microsomal membranes. Figure 4B, also showed that the overexpressed SERCA1‐EGFP membranes were more resistant to inhibition by HBCD, maintaining activity levels of that seen with non‐transfected cell membranes at 3 μm HBCD. Cell viability studies on the transfected versus non‐transfected cells also showed that upon exposure to HBCD for 24 h the SERCA1‐EGFP Ca^2+^ATPase transfected cells were more resistant to cell death as the LC_50_ values had increased from about 4 μm in non‐transfected cells, to 15 μm, in SERCA1‐EGFP transfected cells (Fig. 4C). FACs analysis also pooled the cells into viable versus non‐viable populations (via PI staining), as well as transfected versus non‐transfected cell populations (via the presence of GFP). Figure 4C showed that following 24 h treatment with 3 μm HBCD, four populations of the cells could be determined. Of the two cell populations which had not been effectively transfected with SERCA1‐EGFP, approximately equal numbers of cells were found in the viable versus non‐viable populations. However, in those cells that had been successfully transfected with the SERCA1‐EGFP, about 90% of those cells remained within the viable population (Fig. 4D). Taken together these results indicate that overexpression of SERCA Ca^2+^ ATPases does indeed afford protection against acute toxicity by HBCD.

### Effects of EDC on other Ca^2+^ transporters

Although the main focus has been on the inhibition of SERCA Ca^2+^ ATPases as the main target of acute toxicity by some types of EDC such as BFRs and Alkylphenols, it has been noted that some of these pollutants can also affect other types of Ca^2+^ATPase such as SPCAs and PMCAs as well [51, 55]. It is as yet to be determined whether they also play a major contribution in causing toxicity and cell death.

Another major class of ER Ca^2+^ transporters that occur in cells and that are linked to Ca^2+^ signalling pathways and involved in endocrine function, are the Ca^2+^ channels such as the IP_3_‐sensitive Ca^2+^ channels (IP_3_ receptors), activated by the second messenger, inositol‐1,4,5‐trisphosphate (IP_3_) [59] and the Ryanodine receptor, activated by localised changes in Ca^2+^ concentrations or cADP‐ribose [43]. These Ca^2+^ channels are mainly located on the ER membranes and regulate the release of Ca^2+^ from these Ca^2+^ stores. An early study on the effects of alkylphenols such as nonylphenol on IP_3_ receptors on cerebellar and testis ER membranes indicated that they inhibited Ca^2+^ release induced by IP_3_ in a dose‐dependent manner without affecting the binding of IP_3_ to the channel [60]. This therefore indicated that these chemicals likely affected the channel opening mechanism rather than ligand binding. A more recent study, however, using BPA showed that it could activate the IP_3_ receptors causing intracellular [Ca^2+^] elevation [61], thus indicating that different alkylphenols act upon IP_3_ receptors in different ways. There is limited information with regards to the effects of BFRs on IP_3_ receptors, however, from studies with TBBPA on cerebellar ER microsomes (which are highly enriched in IP_3_ receptors), no effect on the extent of IP_3_‐induced Ca^2+^ release was observed at concentrations up to 6 μm TBBPA [12].

The other major intracellular Ca^2+^ channel found in both muscle and non‐muscle cell ER, called the ryanodine receptor, was shown to be activated by TBBPA [12] and HBCD [27], as determined through their ability to release Ca^2+^ from skeletal muscle SR membranes, which are highly enriched in Ryanodine receptors. The extent to which Ca^2+^ was released by these BFRs was substantially reduced if the membranes were pre‐treated with 1 mM tetracaine, a known Ryanodine receptor inhibitor [12]. Very limited information exists regarding the effects of alkylphenols on the Ryanodine receptors, although some preliminary studies showed that nonylphenol could also cause Ca^2+^ release from SR membrane vesicles [27]. It is known that other man‐made chemicals which could be classed as environmental pollutants such as chloro‐cresol (used in disinfectants), hydroxy‐carbazole (used in the electronics and pharmaceutical industry), are also able to activate the Ryanodine receptor and cause Ca^2+^ release through the ER or SR [62, 63]. Another organelle, which plays a major role in Ca^2+^ homeostasis, metabolic energy production and cell death is the mitochondria. We have shown that some nonylphenols and BFRs can cause a major change in mitochondrial membrane depolarisation [12, 24]. Some more recent studies have implicated that EDCs may also directly affect mitochondria leading to some of the observed metabolically disrupting effects [64].

## Effects of endocrine disruptors on ER stress

**Fig. 5:**
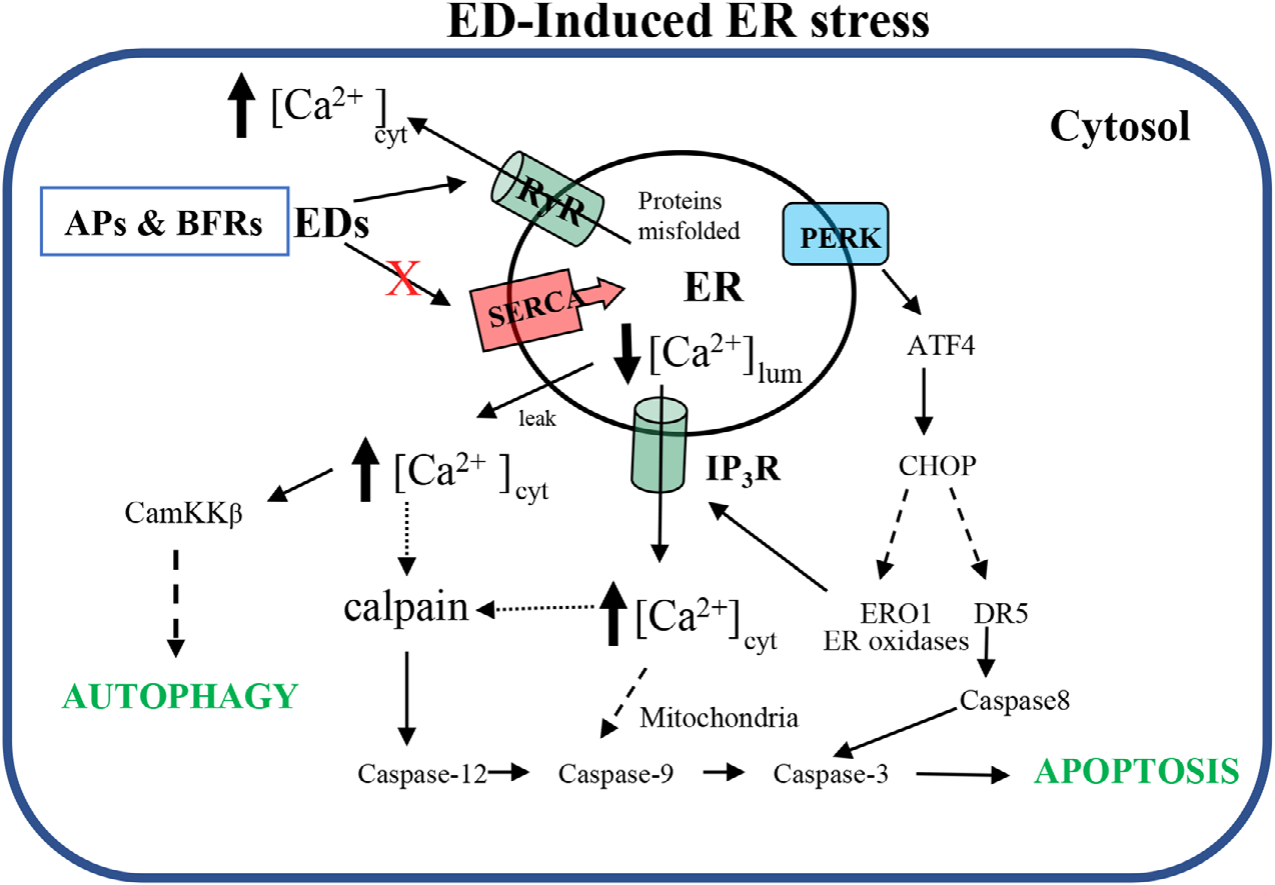
ECD‐induced ER Stress and Ca^2+^‐dependent Cell death pathways. The Figure highlights some of the postulated pathways by which elevation of cytosolic [Ca^2+^] and a concomitant decrease in luminal ER [Ca^2+^] concentrations, caused by EDC can lead to apoptosis and autophagy through activation of both Ca^2+^‐dependent mechanisms in the cytosol and the ER stress response.

From the information presented above, it is clear that some endocrine disruptors, including alkylphenols and BFRs, are able to interact with ER Ca^2+^ transporters to cause Ca^2+^ release from this organelle, leading to elevated cytosolic [Ca^2+^] levels. It is well documented that exaggerated and prolonged increases in cytosolic [Ca^2+^] levels, can hyperactivate a number of Ca^2+^‐dependent enzymes and proteins which can activate cell death pathways, through a variety of mechanisms. Some of these mechanisms can involve other organelles such as the mitochondria leading to cytochrome C release and initiation of intrinsic apoptosis pathway via caspase‐9 [26]. In addition to elevation of cytosolic Ca^2+^ concentration levels and activating Ca^2+^‐dependent cell death pathways, another consequence of this, is depletion of the ER luminal [Ca^2+^] levels, which is known to lead to activation of the unfolded/mis‐folded protein response (UPR) and the initiation of the ER stress response [65]. Therefore, inhibition of SERCA Ca^2+^ ATPase in the ER which leads to depletion of ER Ca^2+^ concentration is likely to be a major contributory factor in ER stress activation. Several studies have recently shown that some BFRs including HBCD can initiate the ER stress response through activation of IRE1 and PERK. The activation of IRE1 was recently confirmed through the use of a fluorescent biosensor which reports on the splicing XBP1 RNA [25], IRE1 is activated as a survival mechanism to try to reduce ER permeabilization and avoid apoptosis during sub‐toxic exposure to chemical insults. To further confirm that the ER stress response is initiated by BFRs, several cell types were preincubated with an ER stress response inhibitor, tauroursodeoxycholic acid (TUDCA), prior to treatment with HBCD. The results showed that the addition of TUDCA afforded substantial protection against cell death [25]. In addition, PERK is also activated by the ER stress response, which causes the phosphorylation of eIF2α, leading to a reduction of ER protein accumulation and translation of the ATF4 and which also increases CHOP expression. Treating cells with HBCD and other BFRs has been shown to increased CHOP expression when cells are treated with HBCD, further implicating the activation of ER stress response [66, 67]. Furthermore, increased CHOP expression controls the expression of other target genes, such as ERO‐1α, which is known to activate the IP_3_ receptor, releasing further Ca^2+^ from the ER, thus contributing to endoplasmic reticulum stress–induced apoptosis [68]. ATF4 is also known to activate the death receptor 5 (DR5) which initiates extrinsic apoptosis through caspase 8 [69]. Figure 5, shows some of the events initiated by elevation of cytosolic [Ca^2+^] levels, and a decrease in luminal ER [Ca^2+^] levels caused by exposure to EDC, like HBCD, that can then lead to cell death through apoptosis and autophagy.

## Conclusions

It is clear from the literature that environmentally pervasive endocrine disrupting chemicals have multiple mechanisms of action. A substantial body of evidence exists to show that many of these EDC are able to bind to oestrogen and other hormone binding receptors, to affect signalling processes that underlie endocrine function, as well as neuronal development and function. The fact that many of these EDCs persist within the environment and bioaccumulate within the food chain can potentially lead us to take up higher amounts than we would normally do, due to direct exposure from low level sources.

Over the last 25 years there is now a growing body of evidence to also show that several types of environmentally present EDC are able to affect Ca^2+^ signalling pathways, causing exaggerated elevations in cytosolic [Ca^2+^] levels, through their action on various intracellular Ca^2+^ transporter proteins. The evidence presented here would favour the view the cytotoxic effects of some EDC in causing cell death would normally occur after acute exposure to these chemicals, rather than prolonged sub‐toxic exposure, which may more likely alter normal physiological developmental processes.

In this review, a considerable body of evidence is presented to indicate that acute cytotoxic exposure by some types of EDC is directly correlated to the inhibition of SERCA Ca^2+^ ATPases and that this is confirmed by the observation that overexpression of SERCA Ca^2+^ ATPases can resist this EDC‐induced cytotoxicity. However, some questions that still need to be addressed, include: what role, if any, do the effects of EDC have on other Ca^2+^ transporters such as other types of Ca^2+^ ATPases (SPCA and PMCA), as well as Ca^2+^ channels (ie IP_3_ receptors and Ryanodine receptors), in contributing to cytotoxicity via elevated of cytosolic [Ca^2+^] levels. Furthermore, as buffering of the elevation of cytosolic [Ca^2+^] using the cytosolic Ca^2+^ chelating agent BAPTA, only partially protects against cell death, a further exploration of the role of decreasing luminal ER [Ca^2+^] and the activation of the ER stress response leading to cell death, needs to be more fully explored and elucidated.

## Conflict of interest

The authors declare no conflict of interest.

## Author contributions

FM originated the concept and wrote the review; NAM was involved in the data analysis and information gathering for the review; BJ was involved in information gathering for the review; MT was involved in information gathering for the review; FA‐M was involved in data analysis and information gathering.
